# Supplementary material for: Application of the JA-CHRODIS Integrated Multimorbidity Care Model (IMCM) to a Case Study of Diabetes and Mental Health
Source: Int J Environ Res Public Health. 2019 Dec 17;16(24):5151. doi: 10.3390/ijerph16245151 (PMC6950036; doi:10.3390/ijerph16245151)
Supplement: Supplementary file 1 [file ijerph-16-05151-s001.pdf]

International Journal of
*Environmental Research and Public Health*

MDPI

**Supplementary Material, File S1**

Case study, describing a hypothetical patient, developed and distributed among experts to assess the applicability of the JA-CHRODIS Integrated Care Model for Multimorbidity (Maria´s case).

Maria is a 71-year-old woman, married, with two children; a son and a daughter. She has completed primary studies and now she is retired (she was a secretary). Maria lives with her husband, five years older than her, who is overweight and suffers from chronic obstructive pulmonary disease (COPD) and mild dementia. Lately, he is increasingly forgetful, and last week he got lost on his way back from the supermarket.

She usually takes care of two small grandchildren and takes them to school. She also takes care of housework when she feels well (on "good days"), while she rests for a long time on "bad days", when she feels tired, sad, and her back pain increases. However, she makes sure that her husband goes to the doctor and takes his medication. She is able to take care of herself, but she has frequently some difficulties in walking due to her back pain.

Maria has type II diabetes, diagnosed 20 years ago and treated with metphormin. She also suffers from hypertension, mild-moderate obesity (body mass index, BMI=32), and chronic low back pain. She is actually taking six different medicines, five times a day. Two years ago, her sister died, and since then she has been teary, avoids going out with her female friends, and her back pain has increased. She has insomnia and sometimes feels restless during the daytime. Now Maria tends to worry about her grandchildren and her husband, and her daughter complains from her pessimistic view of things. She used to enjoy cooking, but now she mostly buys precooked food. Before, she was a vital, cheerful woman, known for her good sense of humor.

She lives in a small flat with her husband, in the second floor with no elevator and several flights of stairs to access the street. Due to her low back pain, she has some problems walking, doing housework, and going outside for shopping. She likes going to church and meeting friends for playing cards and knitting, but lately she has reduced her activity and hobbies.

In the last year, Maria has visited her GP or nurse almost every month, sometimes twice a month, due to several complaints: Shortness of breath, insomnia, fatigue, back pain, dizziness, diabetes, etc. She has been also referred to a specialized care in five occasions, visiting two different specialists; the ophthalmologist and the orthopedic surgeon. Maria also had one admission to hospital due to diabetes complications. She had to visit emergency care once after a short panic attack. Her daughter is very worried about her.

**Supplementary Material, File S2**

Questionnaire developed and distributed among experts to assess the applicability of the JA-CHRODIS Integrated Multimorbidity Care Model components to the case study.

*Component 1: Regular Comprehensive Assessment of Patients*

1. Which professional/s should perform the assessment of Maria?
2. Where should Maria be assessed (primary care centre, specialist office, patient's home, etc.)?
3. What assessment tools could be used to evaluate complexity of conditions/treatment (interview, questionnaires, chart review, electronic medical record, etc.)? Please be specific.
4. What assessment tools would be appropriate and necessary to evaluate Maria's preferences/resources (interview, questionnaires, chart review, electronic medical record, etc.)? Please, be specific.
5. What kind of information should be obtained by the use of the assessment tool?
6. Who should be responsible for providing an individualized care plan for Maria? How frequently should Maria be comprehensively assessed?

*Component 2: Multidisciplinary, Coordinated Team*

1. Who should be part of the multidisciplinary team for the case of Maria? Please list the multidisciplinary healthcare team members that, at minimum, should be involved.
2. What kind of communication tools and ways (common electronic chart, ad hoc meetings, clinical sessions, etc.) among those forming the team should there be to ensure a coordinated care of Maria's health?
3. Who should lead the discussion in the team?

*Component 3: Professional Appointed as Coordinator of the Individualized Care Plan and Contact Person (Case Manager).*

1. How important it is to have a named contact person and/or clinician acting as the primary contact point and coordinating communication between Maria and the team?
2. Which competencies should the contact person have?
3. Which tasks should he/she perform?
4. Should the clinician and the contact person be the same individual?

*Component 4: Individualized Care Plans*

1. Who should be responsible for writing Maria's care plan?
2. What main health outcomes or goals should include Maria's care plan?
3. How frequently should Maria's care plan be revised?
4. How should Maria's care plan be assessed to ensure that the desired outcomes are being met?

*Component 5: Implementation of Evidence Based Practice*

1. Which specific guidelines should be used in Maria's case? Please specify. If there is none available, please indicate.

*Component 6. Training Members of the Multidisciplinary Team*

1. Do you know of any specific training programs for the care team in order to assess Maria's health needs? Please specify.
2. Who should attend this kind of training?
3. Which specific components should have these programs?
4. Should these training programs be continuous or periodical?

*Component 7. Developing a Consultation System to Consult Professional Experts*

1. Which professional experts should be consulted to provide an adequate care to Maria's?
2. When and how frequently should professional experts be consulted?
3. How should the access to experts be provided? Please give examples.

*Component 8. Training of Care Providers to Tailor Self-Management Support Based on Patient Preferences and Competencies*

1. Are there specific training for staff on self-management for Maria's case? Please specify.
2. Who should provide the training?
3. Which staff member(s) should attend the staff self-management training?

*Component 9. Providing Options for Patients and Families to Improve Their Selfmanagement*

1. Which aspects of Maria's health care may be self-managed?
2. Which self-management training format would be more appropriate for Maria?
3. Which family member of Maria should attend training?

*Component 10. Shared Decision Making (Care Provider and Patients)*

1. How should care providers facilitate Maria's involvement in shared decision making?
2. How should care providers facilitate the involvement of Maria's family members in shared decision making?
3. Who should inform Maria about her health conditions and share decisions with her?

*Component 11. Electronic Patient Records and Computerized Clinical Charts*

1. Which kind of information should be registered in Maria's electronic chart to be available for all providers?
2. Who should be responsible for updating information in Maria's electronic patient record?

*Component 12. Exchange of Patient Information (With Permission of Patient) between Care Providers and Sectors by Compatible Clinical Information Systems.*

1. Who should have access to Maria's information?
2. How should confidentiality be protected in Maria's case?
3. Should any information about Maria's history not be shared, for confidentiality issues? Please specify.

*Component 13. Uniform Coding of Patients' Health Problems Where Possible*

1. Should the health care staff use uniform coding and/or classification systems?
2. Which coding and/or classification systems may be used in Maria's case for grouping her diagnoses and other information relating to their treatment and care?

*Component 14. Patient-Operated Technology Allowing Patients to Send Information to Their Care Providers*

1. Does Maria have the capacity to utilize technology?
2. How should staff facilitate her use of technology?
3. Please give some examples of patient-operated technology that Maria could use.

*Component 15. Supporting Access to Community- and Social-Resources*

1. How should staff facilitate Maria's access to community and social resources?
2. Which team member should be responsible for facilitating Maria's access to community and social resources?
3. Which community and social resources would be most helpful for Maria?

*Component 16. Involvement of Social Network (Informal), Including Friends, Patient Associations, Family, Neighbours*

1. Which members of Maria's social network should be involved in her care?
2. How should they be involved?
3. Which team member should be responsible for involving Maria's social network in her care?

**Supplementary material, Figure S1–S5**

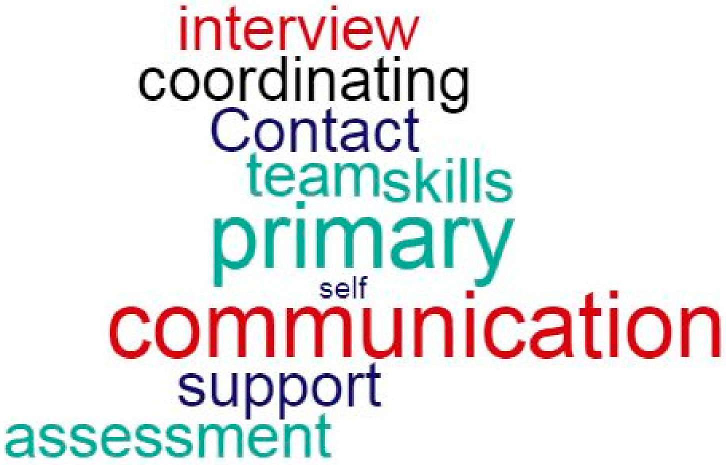


**Figure S1.** Word cloud chart section 1: Delivery of care.

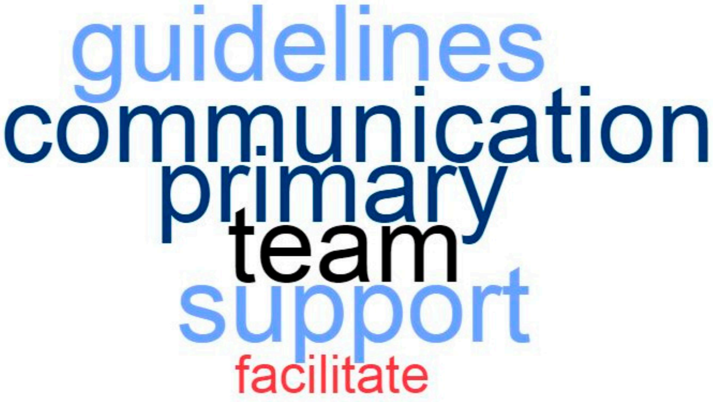


**Figure S2.** Word cloud chart section 2: Decision support.

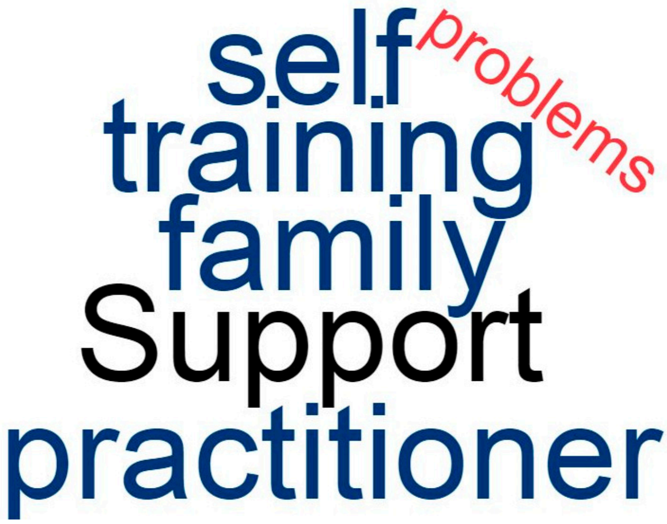


**Figure S3.** Word cloud chart section 3: Self-management support.

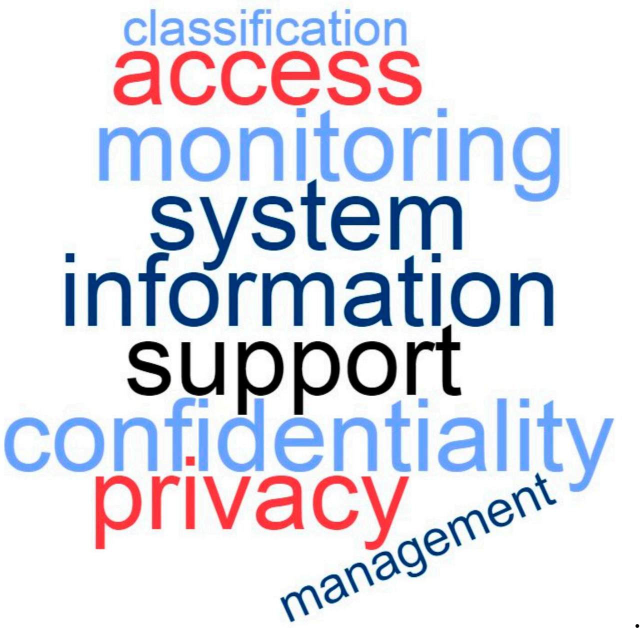


**Figure S4.** Word cloud chart section 4: Information systems and technology.

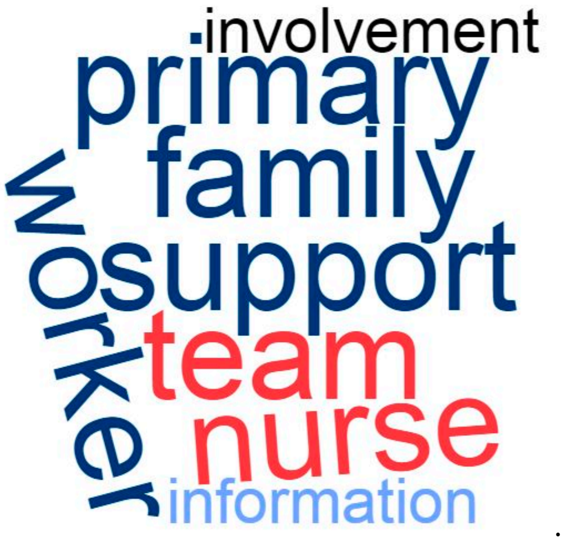


**Figure S5.** Word cloud chart section 5: Social and community resources.
